# Spatiotemporal Variation in Actual Evapotranspiration and the Influencing Factors in Ningxia from 2001 to 2020

**DOI:** 10.3390/ijerph191912693

**Published:** 2022-10-04

**Authors:** Huihui Liu, Dongdong Song, Jinling Kong, Zengguang Mu, Qiutong Zhang, Xixuan Wang

**Affiliations:** 1 College of Geological Engineering and Geomatics, Chang’an University, Xi’an 710054, China; 2 Institute of Natural Resources Survey of Ningxia, Yinchuan 750002, China; 3 School of Public Administration, China University of Geosciences (Wuhan), Wuhan 430074, China; 4 Natural Resources Information Center of Ningxia, Yinchuan 750002, China

**Keywords:** actual evapotranspiration, variation trend, NDVI, meteorological factors, MOD16, future trend

## Abstract

Surface evapotranspiration (ET) is an important part of the hydrological cycle. Based on the MOD16 ET product and the data collected by meteorological stations, this study investigated, for the first time, the characteristics, variation trend and influencing factors of actual ET in Ningxia from 2001 to 2020 along temporal and spatial scales using the Theil–Sen median trend analysis, Mann–Kendall test and Hurst index, and predicted the future trend of ET. The results revealed a strong correlation between the MOD16 ET product and ET data collected at meteorological stations (r = 0.837, R^2^ = 0.701). Over the past 20 years, the annual ET in Ningxia showed an overall increasing trend, and the proportion of the increasing area was 96.58%. Quarterly ET varied over time, with the highest value in the third quarter and the lowest value in the second quarter. Annual ET showed a positive correlation with normalized difference vegetation index (NDVI), surface temperature and precipitation but no correlation with relative humidity. Additionally, the Hurst index revealed areas showing a persistent increase in ET, accounting for 84.91% of the total area, indicating that the future trend of ET in Ningxia is consistent with the past trend.

## 1. Introduction

Actual evapotranspiration (ET), defined as the sum of soil evapotranspiration and vegetation transpiration, is the main index used to evaluate regional surface energy, climate change and water balance. It is a significant part of the water cycle and forms a crucial link between the ecological environment and water resource assessment. Each year, 60% of the land precipitation returns to the atmosphere via ET [1]. The quantity of water resources greatly affect the quality of human agricultural production and livelihood, especially in arid and semi-arid regions. Therefore, accurate estimation and timely management of the spatiotemporal trend and influencing factors of ET are very important and can provide a scientific basis and an important reference point for regional ecological water conservation and protection, as well as the optimization of water resource allocation.

The traditional methods of ET measurement are time-consuming, small-scale, complicated and expensive, and the limited number of field observations do not reflect the spatiotemporal dynamics of ET at the regional scale. However, remote sensing (RS) technology can overcome these limitations as it can obtain ET data over a long time series, on a large scale within a short time and at a relatively low cost. Moreover, the data collected using RS technology is more informative than those collected using traditional methods. 

The MODIS ET project is a part of the NASA/EOS project designed to estimate the global terrestrial ET from the land surface of Earth using satellite RS data. The MOD16 global ET product was produced and released by NASA and amended using the Penman–Monteith (P–M) equation [2]. The MOD16 ET product facilitates the calculation of the water and energy balance, and provides key information on water resource management, especially for ET trend studies. The MOD16-based ET trend has been reported in many studies. Castelli studied the changes in ET over the European Alps based on 20 years of MOD16 data [3]. Dias et al. estimated the monthly reference ET of Brazil [4]. Cheng et al. investigated the spatiotemporal ET patterns in China [5]. The MOD16 ET product has also been used to analyze the trend of ET variation in northwestern Mexico [6], Bulgaria [7], Mongolia [8], China [5,9,10,11], Henan Province [12], Guizhou Province [13], Shanxi Province [14], Xinjiang [15], Yinchuan Plain [16] and Sanjiang Plain [17]. 

With long time-series ET data, the influence of changes in vegetation and climate on regional ET can be quantified. Vegetation links the energy exchange, water cycle and biochemical cycle between the earth and atmosphere. Different vegetation types grow on different underlying surfaces, and vegetation growth affects ET by regulating the land surface roughness, albedo and water interception [18,19]. Climate plays a significant role in the transport of water and heat; therefore, its influence on ET has always interested scholars. In the Nile Basin, ET variability was affected by climate and vegetation [20]. During winter in Mongolia, ET was greatly affected by temperature, normalized difference vegetation index (NDVI) and precipitation during the growing season [21]. Surface vegetation had a significant impact on ET in southeast China [5]. In Sanjiang Plain, the surface ET was greatly influenced by precipitation and vegetation cover [17]. In the Xiliao River Plain, the NDVI and precipitation had different effects on ET from different land types [22]. NDVI, precipitation and temperature were the main factors affecting the actual ET in the Huang-Huai-Hai River Basin [23]. Vegetation cover had a great impact on the ET of grasslands in the Yili Valley [24]. In Henan Province, the NDVI, temperature and precipitation were identified as the main factors affecting the variation in ET [12]. In Guizhou Province, precipitation had the greatest influence on ET variation [13]. 

Water resources play a vital role in arid areas, especially in inland regions such as Ningxia. Lack of rainfall and the shortage of water have always been key in restricting local agricultural production, economic growth and social development. The ecological environment is extremely fragile in Ningxia, which is extremely sensitive to climate and environmental changes. Therefore, studies on the ET trend and the influencing factors are significant in Ningxia. Additionally, Ningxia has only a limited number of observation stations, and the observation data of ET do not fully reflect the overall situation in this region. The use of the MOD16 ET product can fully resolve these problems. Moreover, only a few studies have been conducted on ET in Ningxia in recent years. Thus, it is essential to study the trend and influencing factors of ET in Ningxia based on the MOD16 ET product, which can provide a theoretical basis and scientific foundation for agricultural production; ecological protection; and the rational utilization and development of water resources in arid and semi-arid regions.

In summary, we aimed to: (1) evaluate the accuracy of the MOD16 ET product in Ningxia; (2) estimate the spatiotemporal trend of the change in ET in Ningxia from 2001 to 2020; (3) analyze the influence of NDVI and meteorological factors (surface temperature, precipitation and relative humidity) on ET; and (4) predict the future trend of ET in Ningxia.

## 2. Materials and Methods

### 2.1. Study Area

**Figure 1:**
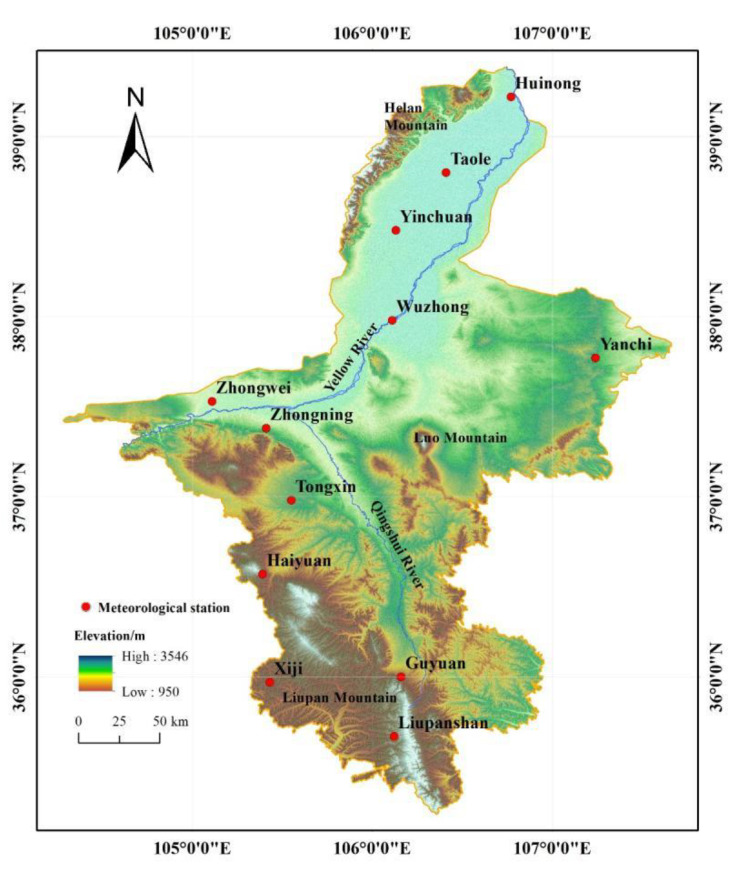
The terrain of Ningxia and the spatial distribution of 12 meteorological stations used in this study. Source: ASTER GDEM 30M (provider: Geospatial Data Cloud website, http://www.gscloud.cn/sources/accessdata/310?pid=302, accessed on 8 May 2022).

The Ningxia Hui Autonomous Region (Ningxia) (Figure 1) is located in the upper reaches of the Yellow River and borders Gansu Province in the south, the Inner Mongolia Autonomous Region in the west and north and Shaanxi Province in the east. It covers a total area of 66,400 km^2^. The terrain from north to south is long and narrow, and the distance is 456 km between north and south and 250 km between east and west. The altitude of Ningxia decreases gradually from south to north with an average altitude of 110 m. It is located in the temperate continental arid and semi-arid area and experiences an average annual temperature of 8 °C and an annual sunshine period of 3000 h, making Ningxia one of the regions receiving the most sun radiation in China. The average annual precipitation in Ningxia is 289 mm and the per capita available water resource is only 603 m^3^, which is only 1/3rd of the national average and far below the warning line of 1700 m^3^ of water shortage in the world [25]. However, the distribution of water resources in Ningxia is extremely uneven. Most of the water resources are distributed in the Yellow River irrigation area, which is located in the north of Ningxia, although the water resources are mainly provided by the Yellow River. The central arid area of Ningxia is the most water deficient, with less surface water and high salt content. In the semi-arid and semi-humid mountainous areas of Ningxia in the south, the water resources are relatively abundant and the river system is well developed. Climate drought, water shortage and uneven distribution of water resources seriously affect the development of local economic growth and social development.

### 2.2. Data Sources and Preprocessing

#### 2.2.1. Remotely Sensed Data

The MOD16 ET product of the study area was obtained from the NASA Goddard Earth Sciences Data and Information Services Center website (https://modis.gsfc.nasa.gov/data/dataprod/mod16.php, accessed on 8 May 2022) and provided information about 8-day global terrestrial evapotranspiration at 500 m spatial resolution. The MODIS reprojection tool (MRT, NASA, Washington, DC, USA) was used to perform the batch processing of projection transformation and band extraction, and the Python program code and ArcGIS software (Esri, Redlands, CA, USA) were used to compute the weighted average of the data to determine the annual ET and quarterly ET.

#### 2.2.2. Meteorological Data and NDVI

The field observations of ET and relative humidity recorded at 12 meteorological stations in the study area were downloaded from the China Meteorological Data Service Centre website (http://data.cma.cn/, accessed on 9 March 2022). To obtain the measured data of meteorological stations at a continuous spatial scale, the abnormal data were removed, and the missing data were supplemented using Kriging interpolation. The spatial interpolation was processed with the ArcGIS software.

Daytime and nighttime surface temperatures were obtained from MOD11A2 at a time resolution of 8 days and spatial resolution of 1000 meters. The MRT was used for data format conversion and projection transformation, Python program code was used for data synthesis and ARCGIS software was used for cutting. The monthly surface temperatures within the study area were obtained using data processing.

Precipitation data were obtained at a spatial resolution of 0.1° × 0.1° from the Integrated Multi-satellite Retrievals for the GPM product, which was downloaded from the NASA GES DISC website (https://disc.gsfc.nasa.gov/, accessed on 20 March 2022). The data were batch-processed using Python and MATLAB program code.

The data of NDVI were obtained from the MOD13 Version 6 product, which provided vegetation index values on a per-pixel basis at 16-day intervals and 500 m spatial resolution. The product was released by NASA (https://modis.gsfc.nasa.gov/data/dataprod/mod13.php, accessed on 15 March 2022). Data format conversion, coordinate conversion, invalid value processing, cutting and resampling were performed using the MRT software, ArcGIS software and Python program code.

### 2.3. Trend Analysis

The ET variation trend in Ningxia was analyzed using the Theil–Sen median trend analysis and Mann–Kendall test, which were generally combined to identify the trend of long-term data.

#### 2.3.1. Theil–Sen Median Trend Analysis

The Theil–Sen median trend analysis is a robust nonparametric statistical method, which did not require the data to obey a certain distribution pattern and reduced the influence of noise [8]. This method has been widely used in the trend analysis of *ET* time-series data at the pixel scale [8,12,16,26,27]. The Theil–Sen estimator (*S_ET_*) indicated the median of the slope of *ET* data combinations and was calculated as follows:

(1) $$S_{ET}=median\left(\frac{ET_{j}-ET_{i}}{j-i}\right),\;2001\leq i\leq j\leq 2020\;$$

*S_ET_* > 0 reflected an increasing ET trend and *S_ET_* < 0 indicated a decreasing *ET* trend.

#### 2.3.2. Mann–Kendall Test

The Mann–Kendall test, developed by Mann [28] and Kendall [29], was used to ascertain the significance of time-series changes. The Mann–Kendall test was insensitive to missing data, independent of data distribution and required fewer assumptions. This method is recommended by the World Meteorological Organization as a nonparametric statistical test and has been widely used in trend testing [8,27,30,31,32,33,34,35,36]. 

The Mann–Kendall statistic (*S*) was calculated as follows:

(2) $$S=\overset{n-1}{\underset{i=1}{\sum }}\overset{n}{\underset{j=i+1}{\sum }}sign\left(ET_{j}-ET_{i}\right)\;\;\;\;\;\;\;\;\;$$

where *ET*_1_, *ET*_2_, *ET*_3_, …, *ET_n_* were the time series of *ET*; $ET_{i}$ and $ET_{j}$ represented the *ET* at times *i* and *j* (*i* < *j*), respectively; *n* was the length of the time series (*n* = 20 in this study); and the results were recorded as $sign\left(ET_{j}-ET_{i}\right)$:

(3) $$sign\left(ET_{j}-ET_{i}\right)=\{\begin{array}{c} 1,\;\;\;\;\;ET_{j}-ET_{i}>0\\ 0,\;\;\;\;\;ET_{j}-ET_{i}=0\\ -1,\;\;\;\;\;ET_{j}-ET_{i}<0 \end{array}$$

The variance of *S* (*Var*(*S*)) was used to estimate the statistical significance of the trend and was computed as:

(4) $$Var(S)=\frac{n(n-1)(2n+5)}{18}$$

The test statistic *Z* was computed as:

(5) $$Z=\{\begin{array}{c} \frac{S-1}{\sqrt{Var\left(S\right)}},\;\;S>0\\ 0,\;\;\;\;\;\;\;\;\;\;\;\;\;S=0\\ \frac{S+1}{\sqrt{Var\left(S\right)}},\;\;S<0 \end{array}$$

*Z* is assumed to follow a standard normal distribution. If *Z* is greater than a critical value of *Z* 1 − α/2, then the trend is accepted as statistically significant, based on the statistical significance level of 1 − α/2, where α is generally taken as 0.01 (1%), 0.05 (5%) or 0.01 (1%). The sign of the statistic, *S*, signifies a positive or a negative monotone trend.

### 2.4. Correlation Analysis

In this study, the Pearson correlation coefficient (*r*) and the determination coefficient (*R*^2^) were applied to analyze the correlation between MOD16 ET and the field ET. Additionally, *r*, which generally indicates the correlation between dependent and independent variables, was also used to analyze the correlation of MOD16 ET with key meteorological factors and NDVI. *R*^2^ was used as a measure of the quality of fitting, which reflected the proportion of variability explained by the fitted model [6]. The values of r and *R*^2^ ranged between −1 and 1, and were calculated with the SPSS software (IBM, United States) using the following formulae:

(6) $$r=\frac{\sum_{i=1}^{n}\left(x_{i}-\bar{x}\right)\left(y_{i}-\bar{y}\right)}{\sqrt{\sum_{i=1}^{n}{\left(x_{i}-\bar{x}\right)}^{2}\cdot \sum_{i=1}^{n}{\left(y_{i}-\bar{y}\right)}^{2}}}$$

(7) $$R^{2}={\left[\frac{\sum_{i=1}^{n}\left(x_{i}-\bar{x}\right)\left(y_{i}-\bar{y}\right)}{\sqrt{\sum_{i=1}^{n}{\left(x_{i}-\bar{x}\right)}^{2}\cdot \sum_{i=1}^{n}{\left(y_{i}-\bar{y}\right)}^{2}}}\right]}^{2}$$

where $\overset{n}{\underset{i=1}{\sum }}\left(x_{i}-\bar{x}\right)\left(y_{i}-\bar{y}\right)$ and $\sqrt{\sum_{i=1}^{n}{\left(x_{i}-\bar{x}\right)}^{2}\cdot \sum_{i=1}^{n}{\left(y_{i}-\bar{y}\right)}^{2}}$ represented the covariance and standard deviation, respectively, between variables *x* and *y*, and *n* was the length of the time series.

### 2.5. Tendency Forecast

In this study, the future tendency of ET was forecasted using the Hurst exponent (H), which quantitatively described the sustainability characteristics of the time-series data and was proposed by Hurst in the analysis of hydrological data obtained from the Nile River [31,37]. The Hurst exponent was calculated via the rescaled range (R/S) analysis, which is briefly described as follows:

For {ET(t)},  (t=1,2,3,…,N), the mean of the time sequence was:

(8) $${\bar{ET}}_{\left(\tau \right)}=\frac{1}{\tau }\;\overset{\tau }{\underset{t=1}{\sum }}{\mathrm{ET}}_{\left(\tau \right)}\;\;\;\;\;\;\;\;\;\;\left(\tau =1,2,\ldots ,n\right)\;$$

The cumulative deviation was:

(9) $$X_{t,\tau }=\overset{t}{\underset{t=1}{\sum }}(ET_{\left(t\right)}-{\bar{ET}}_{\left(\tau \right)})\;\;\;\;\;\;\;\;\;\;\left(1\leq t\leq \tau \right)$$

The range sequence was:

(10) $$R_{\tau }=\underset{1\leq t\leq \tau }{max}X_{t,\tau }-\underset{1\leq t\leq \tau }{min}X_{t,\tau }\;\;\;\;\;\;\;\;\;\left(\tau =1,2,\ldots ,n\right)$$

The standard deviation sequence was:

(11) $$S_{\tau }={\left[\frac{1}{\tau }\;\overset{t}{\underset{t=1}{\sum }}{\left(ET_{\left(t\right)}-ET_{\left(\tau \right)}\right)}^{2}\right]}^{\frac{1}{2}}\;\;\;\;\;\;\;\;\;\left(\tau =1,2,\ldots ,n\right)$$

The Hurst exponent calculation was:

(12) $$\frac{R_{\tau }}{S_{\tau }}={\left(c\tau \right)}^{H}$$

where *c* was a constant. When $\frac{R_{\tau }}{S_{\tau }}\propto \tau^{H},\;$the time series exhibited sustainability characteristics. 

The value of H ranged from 0 to 1 and represented the change in trend with time; 0.5 < *H* < 1 indicated that the process had sustainability characteristics and the future trend was consistent with the past trend; *H* = 0.5 indicated that the future trend was random and no long-term correlation existed; 0 < *H* < 0.5 indicated that the future trend was opposite to the past trend [31,38,39]. The closer the value of *H* was to 1, the greater the likelihood that the past and future trends were similar; the closer the value of *H* was to 0, the greater the likelihood that the past and future trends were opposite.

## 3. Results

### 3.1. Evaluation of MOD16

**Figure 2:**
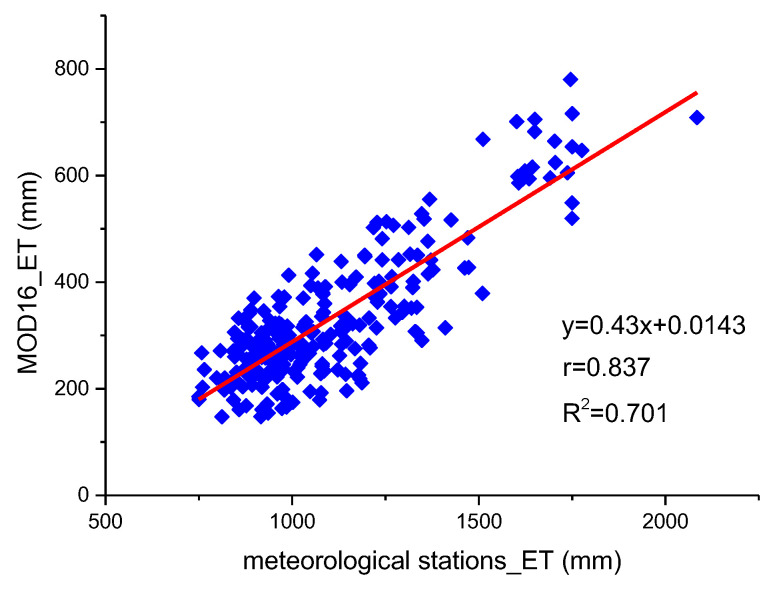
Correlation between MOD16 ET and meteorological station ET data. The red line represents the best fitting line. The point represents the actual ET of 12 meteorological stations from 2001 to 2020.

This study evaluated the accuracy of the MOD16 ET product by analyzing the correlation between the measured ET data obtained from the meteorological stations and the actual ET of MOD16 in Ningxia from 2001 to 2020. The actual ET data of 12 meteorological stations in Ningxia were extracted from the MOD16 ET product, and a correlation analysis was conducted between the actual ET of MOD16 and the measured ET of each meteorological station (Figure 2). The result revealed a significant correlation between the actual ET and the measured ET (*p* < 0.01, r = 0.837, R^2^ = 0.701), indicating that the MOD16 ET product exhibited a good spatiotemporal correlation with the ET of meteorological stations, and the data accuracy met the needs of the surface ET research in Ningxia. Therefore, the MOD16 ET product could be used to study the spatiotemporal distribution characteristics of surface ET and the influencing factors in Ningxia.

### 3.2. Trend of Spatiotemporal Variation in Annual ET

**Figure 3:**
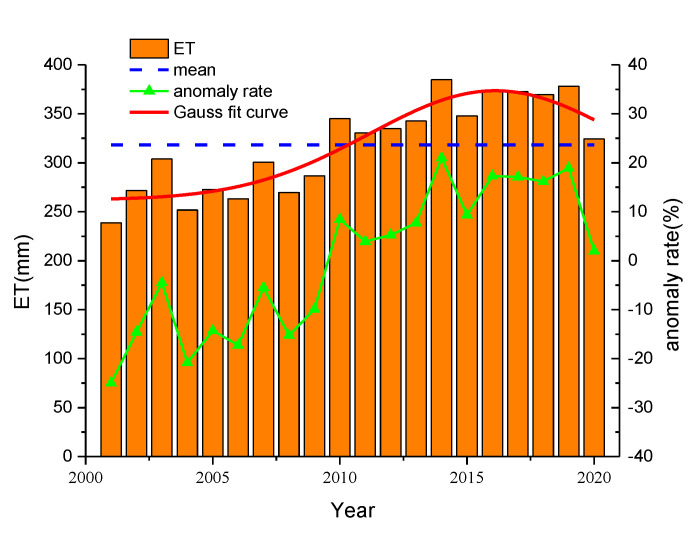
The trend of annual ET in Ningxia from 2001 to 2020.

The temporal variation in annual ET in Ningxia is shown in Figure 3. Between 2001 and 2020, the annual ET in Ningxia fluctuated greatly within the range of 238.67–384.94 mm and fitted with the Gauss curve, which showed an upward trend. The average ET was 318.15 mm and the anomaly rate ranged from −24.98% to 20.99%. The year of 2010 marked a turning point because annual ET values from 2001 to 2009 were below the average ET and annual ET values from 2010 to 2020 were above the average ET. The highest ET was in 2014 and the lowest was in 2001.

**Figure 4:**
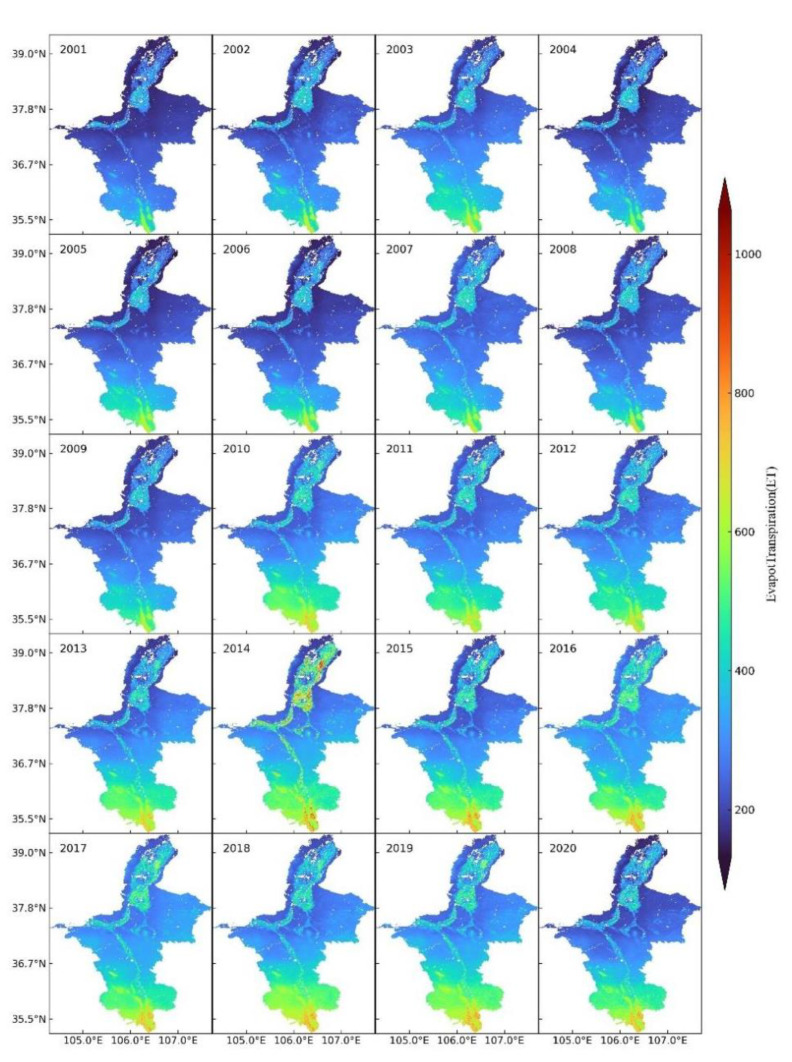
Spatial distribution of annual ET in Ningxia from 2001 to 2020.

Figure 4 shows the spatial distribution of surface ET in Ningxia from 2001 to 2020. In general, the annual ET gradually increased. The most obvious changes in ET were observed in regions along the Yellow River and in southern Ningxia. Since 2010, the increasing range of ET expanded in the southern region, and the highest ET had always appeared in the south part of the southern region. In the northern and central regions, ET along the Yellow River and its tributaries (Qingshui River) was higher than that in the other inland areas. In the mid-eastern region, the annual ET in the area surrounding Luo Mountain also increased each year.

### 3.3. Trend of Spatiotemporal Variation in Quarterly ET

**Figure 5:**
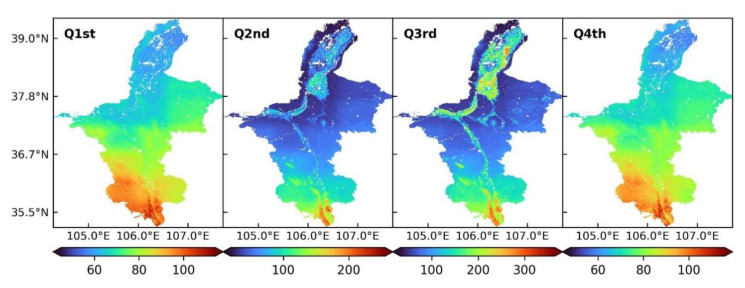
Spatial distributions of quarterly ET in Ningxia from 2001 to 2020.

The annual ET showed a clear quarterly difference in Ningxia from 2001 to 2020. Based on MOD16 ET, the spatial and temporal variation characteristics of quarterly ET were analyzed (Figure 5). Average ET was the highest (104.46 mm) in the third quarter, when higher vegetation coverage and temperature and more precipitation favored the increase of ET. The values of average ET were 76.66, 74.51 and 62.52 mm in the fourth, first and second quarters, respectively. The first and fourth quarters showed similar spatial distribution patterns of ET, which decreased from the south to the north. In the second and third quarters, the ET was higher in southern and northern regions located along the Yellow River and lower in central regions and Helan Mountain. In the second and third quarters, ET along the Yellow River and its tributaries was higher than that in the other inland areas. 

### 3.4. Trend of Spatial Variation in ET

**Figure 6:**
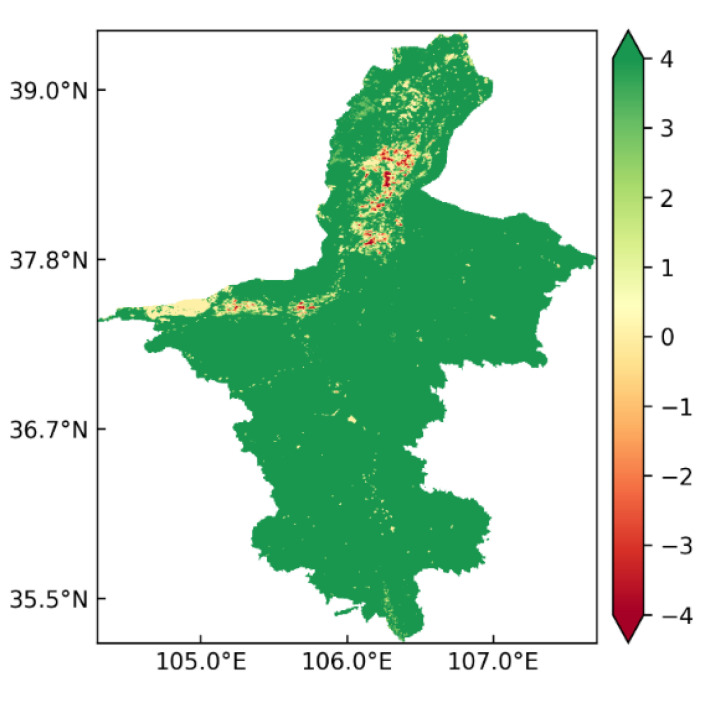
Spatial distribution of ET trends in Ningxia from 2001 to 2020.

The Theil–Sen median trend analysis and Mann-Kendall test were used to analyze the trend of spatial variation in annual ET in Ningxia from 2001 to 2020. The values of the Theil–Sen median were classified as follows: increasing trend (*S_ET_* > 0), no change (*S_ET_* = 0) and decreasing trend (*S_ET_* > 0). Table 1 shows the result of the significance analysis and explains the significance of the trend of spatial variation in annual ET in the paper. Based on a confidence level of 0.05, the Mann-Kendall test results were divided into nine grades: extremely significant increase, significant increase, slight increase, insignificant increase, no change, insignificant decrease, slight decrease, significant decrease and extremely significant decrease. ET showed an increasing trend in the most regions of Ningxia, accounting for 96.58% of the total area (Table 1 and Figure 6). An extremely significant increase in ET was observed in 93.12% of the total area. Approximately 2.24% of the total area, mainly in the Shapotou Desert which is a part of the Tengger Desert and is located in the mid-west region of Ningxia near Inner Mongolia, showed no change in ET. The decreasing trend of ET was detected in 1.18% of the total area, mainly in the urban agglomeration and ecological conservation belt along the Yellow River. The decrease in ET was greatly affected by factors related to human activity, for example, soil erosion caused by continued urbanization and large-scale deforestation. Overall, the annual ET showed an increasing trend in Ningxia from 2001 to 2020.

### 3.5. Correlation between ET and NDVI

**Figure 7:**
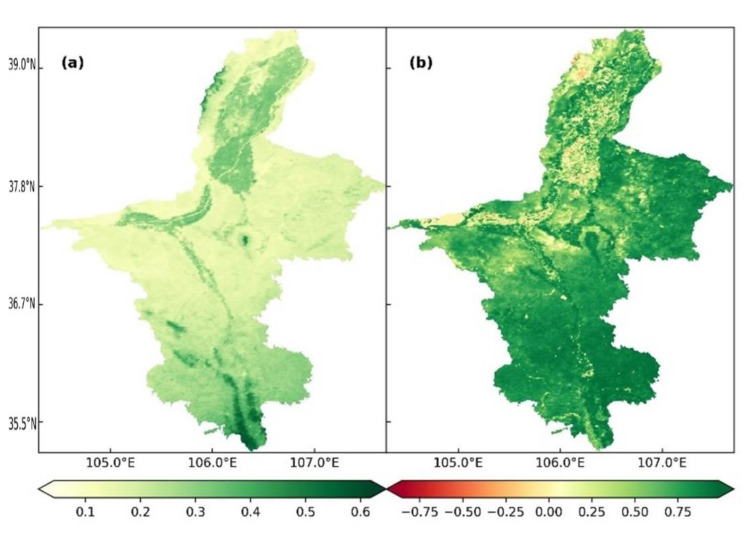
(**a**) Spatial distribution of the average NDVI in Ningxia from 2001 to 2020. (**b**) Spatial distribution of correlation between ET and NDVI in Ningxia from 2001 to 2020.

The correlation between ET and NDVI was analyzed to determine whether NDVI affected ET in Ningxia from 2001 to 2020. As shown in Figure 7a, the average NDVI from 2001 to 2020 was 0.21 (range = 0.43–0.62). The correlation between ET and NDVI varied from −0.85 to 0.98 (average r = 0.684) (Figure 7b). The area of positive correlation between ET and NDVI accounted for 91.63% of the total area, and the region with significant correlation (*p* < 0.5) accounted for 87.05% of the total area. Together, these results suggested that NDVI had a significant positive effect on the annual ET in Ningxia from 2001 to 2020.

The spatial distribution of correlation between ET and NDVI was different for different underlying surfaces. The uneven underlying surface determined the vegetation type, growth and coverage, and thus greatly affected the spatial distribution of ET. Regions showing a significant correlation between ET and NDVI were mainly concentrated in the southern mountainous areas and central and eastern regions, which were mainly covered by woodlands and grasslands. Helan Mountain in the north and the Shapotou Desert in the mid-west were sparsely vegetated, and regions along the Yellow River were mainly cultivated land and cities; in these regions the correlation between ET and NDVI was weak.

### 3.6. Correlation between ET and Meteorological Factors

The variation in ET was closely related to the distribution of water and heat, and the transfer of water and heat were greatly influenced by climate change. Surface temperature and sunshine hours represented the heat condition; precipitation and relative humidity represented the water condition; and average wind speed represented the dynamic condition [40]. The characteristics of ET variation showed that the heat and water conditions greatly affected the temporal and spatial trends of ET in Ningxia. Therefore, surface temperature, precipitation and relative humidity were selected as the main meteorological factors affecting the annual ET in Ningxia from 2001 to 2020.

**Figure 8:**
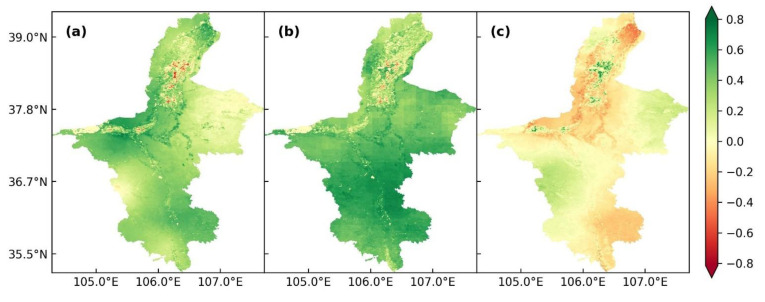
Analysis of the spatial distribution of the correlation between ET and three meteorological factors in Ningxia from 2001 to 2020. (**a**–**c**) Spatial distribution of the correlation between ET and surface temperature (**a**), precipitation (**b**) and relative humidity (**c**).

The spatial distribution of correlation coefficients between ET and three meteorological factors (surface temperature, precipitation and relative humidity) are shown in Figure 8a–c. The annual ET was positively correlated with surface temperature and precipitation in most regions but showed no relationship with relative humidity in Ningxia from 2001 to 2020. As shown in Figure 8a, the correlation coefficient between ET and surface temperature ranged from −0.81 to 0.80, and the average correlation coefficient was 0.304. The positive correlation area accounted for 57.81% of the total area, and the areas showing significant positive correlation (*p* < 0.05) accounted for 24.42% of the total area. The positive correlation areas were mainly distributed in the mid-west, northeast regions and most areas of the southern region. These regions were more affected by surface temperature than the other meteorological factors. The correlation coefficient between ET and precipitation varied from −0.64 to 0.80 (average r = 0.457) (Figure 8b). The positive correlation area accounted for 87.14% of the total area, while the significant positive correlation area (*p* < 0.05) accounted for 63.30% of the total area. The positive correlation areas were mainly concentrated in the southern region, which was affected by precipitation more than the northern and central regions. In the irrigated area along the Yellow River and the Qingshui River, the negative or weak correlation between ET and surface temperature and precipitation had a banding distribution, which was related to the distribution of aridity and irrigation. The irrigated area was located in the arid and semi-arid region with low rainfall and the land was mostly cultivated. In the past 20 years, ET changed little, while the surface temperature and precipitation increased in the irrigated area, so ET was not highly correlated with surface temperature and precipitation. The correlation coefficient between ET and relative humidity varied from zero to one (average r = −0.047) (Figure 8c). The positive correlation area was 1.22% of the total area, and the significant positive correlation area (*p* < 0.05) was only 0.63%. Negative correlation was detected in 3.05% of the total area, and the significant negative correlation area was only 0.35%. Thus, the amount and distribution of relative humidity had no major effect on ET.

### 3.7. Future Trend Predication

**Figure 9:**
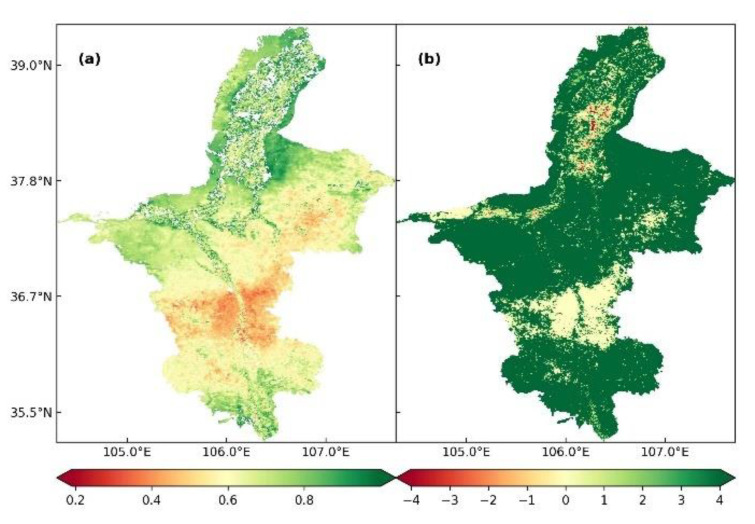
(**a**) Spatial distribution of the Hurst index (H) in Ningxia. (**b**) Future trend of ET in Ningxia.

The Hurst index (H) was used to predict the future trend of ET in Ningxia. H was calculated pixel-by-pixel, according to the raster image of ET (in Ningxia from 2001 to 2020) and the R/S analysis, and a spatial distribution figure of *H* was generated (Figure 9a). The results showed that H varied from 0.19 to 0.99, and the average *H* was 0.66. Areas with *H* > 0.5 accounted for 89.84% of the total area, while those with *H* < 0.5 represented 10.16% of the total area; no areas showed *H* = 0.5. These results indicated that the positive persistence of ET in Ningxia was significant. In other words, the future trend of ET in Ningxia was consistent with the past trend.

To reveal the future trend features of ET, the results of the variation trend of ET (Figure 6) and the Hurst index (Figure 9a) were overlayed and analyzed. The coupled results are shown in Figure 9b and the statistics are shown in Table 2. Areas showing persistent increase in ET accounted for 84.91% of the total area, while those showing an extremely significant persistent increase in ET accounted for 82.16% of the total area and were mainly distributed in most of the northern region, north-central region and southern region. The proportion of area showing a persistent decrease in ET was only 1.04% of the total area, and that showing an extremely significant decrease (mainly in the northern and central regions along the Yellow River) was 0.19%. The regions accounting for 14.05% of the total area could not predict the future trend of ET, specifically in the Shapotou Desert, the south-central region and part of the east-central region; ET should be a continuous concern in these regions.

## 4. Discussion

### 4.1. Principal Findings and Comparison with other Studies

The accurate estimation of ET, timely understanding of spatiotemporal variation characteristics of ET and its influencing factors play an important role in ecological water conservation and protection and the optimal allocation of water resources in arid and semi-arid areas of northwest China. Therefore, this study focused on the temporal and spatial trends of annual ET and quarterly ET from 2001 to 2020 based on the time-series MOD16 data and the influencing factors of ET in Ningxia, and also predicted the future trend of ET. 

In this study, the results showed an increasing trend in annual ET in most areas of Ningxia from 2001 to 2020, and annual ET changed by a larger amount in the southern region than in the northern and central regions, which was mainly related to the topography of the area and ecological restoration projects. Liupan Mountain is located in the south of Ningxia, and its unique geographical location and huge ecological function have a great impact on the local climate, as well as vegetation diversity and coverage. Additionally, the long-term ecological restoration projects enhanced local soil and water conservation, greatly improved the water quality and contributed to the local climate and vegetation. Because of the increase in temperature, precipitation and vegetation coverage [41,42,43], ET stabilized at a high level. Annual ET also varied greatly along the Yellow River and the Qingshui River, which was mainly due to the sufficient water provided by irrigation for ET. In the other areas of the northern and central regions, the ET also showed an overall increasing trend, but it was not obvious, which was related to the lower density and coverage of vegetation, less precipitation and lower temperature.

The proportion of area with an increasing trend of ET reached 96.58%, and that with an extremely significant increasing trend covered 93.12% of the total area, indicating that the annual ET showed an overall increasing trend in Ningxia from 2001 to 2020. This result was basically consistent with other scholars. Yang et al. [10] and Yang et al. [44] discussed the spatiotemporal trend of annual ET in China. In the former study, the annual ET showed an overall upward trend from 2003 to 2016, and a similar result was obtained from 2001 to 2018 in the latter study. Using the SEBS model, Ma [45] estimated the annual ET and reported an increasing trend of ET from 2001 to 2015 in Yinwei Plain, which is located in the mid-north of Ningxia. Cao et al. [46] reported an increasing trend of ET from 2000 to 2017 in the Three River Source area located in the southern region of Ningxia, with the highest ET in Liupan Mountain. Xue et al. [47] investigated the annual actual ET of the Yellow River economic zone in Ningxia based on the MODIS and SEBS model and showed that ET gradually increased from 2001 to 2014.

In the present study, we investigated the effects of NDVI, surface temperature, precipitation and relative humidity on ET; although the former three factors had a significant effect on ET, relative humidity had no obvious effect. We compared our results with those of other studies. Jing et al. [48] showed that temperature, precipitation and relative humidity had positive effects on ET in China from 2000 to 2019. Wang et al. [49] considered temperature, precipitation and relative humidity as the influencing factors of ET in the Loess Plateau in the Shaanxi-Gansu-Ningxia region from 2000 to 2012. The provinces of Qinghai and Ningxia exhibited similar geographical locations (northwest China), climates and environmental conditions. Song et al. [21] identified temperature, precipitation and NDVI as the dominant factors affecting the change in ET in Qinghai Province over the past 20 years. Gong et al. [50] showed a strong correlation between ET and precipitation but a weak correlation between ET and temperature in Ningxia from 2000 to 2014. Wang et al. [16] concluded that temperature, precipitation and relative humidity influenced ET in Yinchuan Plain, which is located in the middle of Ningxia, and ET for desert area land was lower than that for vegetation-covered land, indirectly indicating that NDVI affected the variation in ET. It was shown from above studies that temperature, precipitation and relative humidity always had great effects on ET, but in this study, the effect of relative humidity was different. This was mainly caused by two reasons. First, the influencing factors of ET could have been different in different study areas. Second, the relative humidity was low in the arid and semi-arid regions and the small changes in relative humidity had so little influence on ET that it was not a decisive factor in the change in ET.

### 4.2. Limitations and Future Research Direction

The traditional measurement methods of ET cost more and take much more time than RS technology. Moreover, the ET process is complex, which is why monitoring the spatiotemporal variation in surface ET remains difficult. RS ET products are vital data sources for studying the spatiotemporal variation in ET on regional scales, such as GRACE [9,51], SSEBop [52,53], GLEAM [10,52], LSA-SAF [54,55], BESS [50,56], ETWatch [9,57], ETMonitor [9,58], etc. However, data sources vary in resolution and quality, and the assessments of ET variation are different. Therefore, there are many products that can be applied to the study of ET, but the applicability of different products in different regions needs to be determined through further tests. In follow-up research, ET products should be compared to evaluate their consistencies and uncertainties regarding ET variation, with the aim to obtain more reliable results.

This study performed an in-depth analysis of the effect of NDVI and meteorological factors (surface temperature, precipitation and relative humidity) on the change in ET. However, there are many factors that directly or indirectly affect the change in the actual ET, such as climate, human activities, land use pattern and vegetation type. Therefore, future studies should consider the influence of multiple factors on ET, to provide a theoretical basis for optimizing water resource utilization and management, thus, promoting ecological restoration and further improving agricultural and animal husbandry productivity.

## 5. Conclusions

This study evaluated the accuracy of the MOD16 ET product by analyzing the correlation with the ET measured at meteorological stations. The spatiotemporal characteristics of annual and quarterly ET in Ningxia from 2001 to 2020 were studied using the Theil–Sen median trend analysis and Mann–Kendall test. The effects of NDVI and three meteorological factors (surface temperature, precipitation and relative humidity) on annual ET were also analyzed. Finally, the future trend of ET in Ningxia was predicted using the Hurst exponent. The conclusions of this study are as follows:

1. A correlation analysis was conducted between the ET measured at meteorological stations and the actual (MOD16) ET. The Pearson correlation coefficient was 0.837 and the determination coefficient was 0.701, which indicated that the MOD16 ET product had a good spatiotemporal correlation with the measured ET and its accuracy could fulfill the requirements of surface ET studies in Ningxia.
2. From 2001 to 2020, the annual ET in Ningxia fluctuated between 238.67 and 238.67 mm and the overall ET showed an upward trend with the Gauss curve. The average ET was 318.15 mm and the anomaly rate fluctuated from −24.98% to 20.99%. The highest ET (384.94 mm) was observed in 2014 and the lowest ET (238.67 mm) was detected in 2001.
3. The spatial distribution pattern of the annual ET in Ningxia from 2001 to 2020 showed that the annual ET increased each year but decreased in a stepwise pattern from south to north. In the southern region, the annual ET was always high and showed an upward trend which was mainly caused by the Liupan Mountain and ecological restoration projects. Ecological improvements led to the increase in the surface temperature, precipitation and vegetation coverage. Moreover, the annual ET along the Yellow River and the Qingshui River was significantly higher than that in other inland areas.
4. The average ET of each quarter varied between 2001 and 2020. The third quarter showed the highest average ET, followed by the fourth quarter, first quarter and lastly the second quarter. Moreover, the spatial distribution of ET in the first quarter was similar to that in the fourth quarter, and the spatial distribution of ET in the second quarter was close to that in the third quarter.
5. According to the Theil–Sen median trend analysis and Mann–Kendall test, 96.58% of the total area showed an increasing trend of ET, and 93.12% of the total area showed an extremely significantly increasing ET trend. The region with no variation in ET accounted for 2.24% of the total area and was mainly distributed in the Shapotou Desert. The area showing decreasing ET accounted for only 1.18% of the total area and was mainly affected by human activity-related factors such as soil erosion caused by the expansion of urbanization scope and large-scale destruction of vegetation. In general, the annual ET showed an increasing trend in Ningxia from 2001 to 2020.
6. A positive correlation was detected between ET and NDVI (r = −0.85 to 0.98), and the average correlation coefficient was 0.684. The positive correlation area was 91.63% of the total area and was mainly concentrated in the southern mountainous areas and the central and eastern regions. A weak correlation was detected between ET and NDVI in Helan Mountain, the Shapotou Desert and in regions along the Yellow River. The spatial difference in the correlation between ET and NDVI was caused by the unevenness of the underlying surface, which resulted in different vegetation types and coverage.
7. Surface temperature, precipitation and relative humidity were selected as the main meteorological factors for analyzing their effects on the annual ET. By analyzing the spatial distribution of correlation coefficients between ET and the three meteorological factors, we found that annual ET was positively correlated with surface temperature and precipitation in most regions but showed no correlation with relative humidity.
8. The proportion of area with H greater than 0.5 was 89.84%, and with H less than 0.5 was 10.16%, indicating that the future trend of ET in Ningxia was consistent with the past trend. We overlaid the spatial distribution patterns of the variation trends of ET and H and found that areas with an extremely significant persistent increase in ET accounted for 82.16% of the total area, mainly in most of the northern region, the north-central region and southern region. The proportion of the area showing a persistent decline in ET was only 1.04%. No future trend of ET could be determined for 14.05% of the total area, which was mainly distributed in the Shapotou Desert, the south-central region and part of the east-central region. ET should, therefore, be continuously monitored in these regions.

## Figures and Tables

**Table 1:** Statistics of ET trends in Ningxia from 2001 to 2020.

S_ET_ Value	Z	Trend Type	Trend Features	Area (%)
More than 0	|Z| ≥ 2.58	4	Extremely significant increase	93.12
	1.96 ≤ |Z| < 2.58	3	Significant increase	1.65
	1.645 ≤ |Z| < 1.96	2	Slight increase	0.47
	|Z| < 1.645	1	Insignificant increase	1.34
0	Z = 0	0	No change	2.24
Less than 0	|Z| < 1.645	−1	Insignificant decrease	0.69
	1.645 ≤ |Z| < 1.96	−2	Slight decrease	0.10
	1.96 ≤ |Z| < 2.58	−3	Significant decrease	0.19
	|Z| ≥ 2.58	−4	Extremely significant decrease	0.20

**Table 2:** Statistics of ET trends and the Hurst index (H).

S_ET_ Value	Z	H	Trend Type	Trend Features	Area (%)
More than 0	|Z| ≥ 2.580	>0.5	4	Extremely significant persistent increase	82.16
	1.96 ≤ |Z| < 2.58		3	Significant persistent increase	0
	1.645 ≤ |Z| < 1.96		2	Slightly persistent increase	1.55
	|Z| < 1.645		1	Insignificant persistent increase	1.19
Less than 0	|Z| < 1.645	>0.5	−1	Insignificant persistent decrease	0.59
	1.645 ≤ |Z| < 1.96		−2	Slightly persistent decrease	0.09
	1.96 ≤ |Z| < 2.58		−3	Significant persistent decrease	0.17
	|Z| ≥ 2.58		−4	Extremely significant persistent decrease	0.19
**—**	—	<0.5	0	Unidentified	14.05

## Data Availability

Not applicable.
